# Disease severity and proton pump inhibitor use impact strongest on faecal microbiome composition in liver cirrhosis

**DOI:** 10.1111/liv.14382

**Published:** 2020-01-24

**Authors:** Vanessa Stadlbauer, Irina Komarova, Ingeborg Klymiuk, Marija Durdevic, Alexander Reisinger, Andreas Blesl, Florian Rainer, Angela Horvath

**Affiliations:** ^1^ Division of Gastroenterology and Hepatology Department of Internal Medicine Medical University of Graz Graz Austria; ^2^ Center for Biomarker Research in Medicine (CBmed) Graz Austria; ^3^ Center for Medical Research Medical University of Graz Graz Austria; ^4^ Institute of Pathology Medical University of Graz Graz Austria; ^5^ Intensive Care Unit Department of Internal Medicine Medical University of Graz Graz Austria

**Keywords:** aetiology, cirrhosis, disease severity, inflammation, malnutrition, proton pump inhibitor

## Abstract

**Background & Aims:**

Compositional changes of the faecal microbiome in cirrhosis are well described and have been associated with complications and prognosis. However, it is less well known, which disease or treatment‐related factors affect microbiome composition most distinctively.

**Methods:**

16S rDNA sequencing data of 88 cirrhotic outpatients were investigated. Factors influencing microbiome composition were analysed by univariate and multivariate redundancy analysis. The association of the identified factors with changes in diversity and taxonomic composition was studied in depth using analysis of composition of microbiome, LDA‐effect size and least absolute shrinkage and selection operator regularized regression.

**Results:**

Disease severity and aetiology, proton pump inhibitor (PPI) use, nutritional status, age and C‐reactive protein are significant explanatory variables for faecal microbiome composition in liver cirrhosis. Despite some taxonomic overlaps especially between disease severity and PPI use, we could show that the effects of disease severity, aetiology, PPI use and age are independent factors influencing microbiome composition also in subgroup analyses.

**Conclusion:**

Our cross sectional system biology study identifies disease severity, aetiology, PPI use and age as independent factors that influence microbiome composition in liver cirrhosis. In chronic diseases with high morbidity, such as liver cirrhosis, precise patient metadata documentation is of utmost importance in microbiome analysis. Further studies with a higher sample size are necessary to validate this finding.

Trial Registration Number: NCT01607528

**Abbreviations**
ANCOM: analysis of composition of microbiome
CRP: C‐reactive protein
DAO: diamino‐oxidase
LASSO: least absolute shrinkage and selection operator
LBP: lipopolysaccharide binding protein
LEfSe: LDA‐effect size
LPS: lipopolysaccharide
MELD: model for end‐stage liver disease
PPI: proton pump inhibitor
RDA: redundancy analysis
sCD14: soluble CD14
sCD163: soluble CD 163
SGA: subjective global assessment
sMR: soluble Mannose receptor
TNF: tumour necrosis factor

## 1 INTRODUCTION

Liver cirrhosis is an increasingly common disease with high complication rates. It leads to reduced quality of life and a high burden of disease for patients, their family and the healthcare system.^1^ Cirrhosis is associated with changes in the structure and functionality of the microbiome of the gut^2^, ^3^, ^4^, ^5^ and other body sites, such as skin or the mouth.^6^ Compared to healthy individuals, a decrease in faecal microbial diversity and an imbalance between commensal and pathogenic taxa is seen in patients with cirrhosis. Furthermore, cirrhosis‐associated dysbiosis goes hand in hand with increased gut permeability, intestinal bacterial translocation, intestinal and systemic inflammation leading to complications of cirrhosis and an increased mortality.^3^, ^7^, ^8^, ^9^, ^10^, ^11^ Dysbiosis in general and especially in cirrhosis may be caused by host and environmental factors, which shape the microbiome. These factors include alcohol consumption, aetiology and severity of liver disease, diet composition and medication. 5, 12, 13, 14, 15, 16, 17, 18 Drug intake has emerged as one of the most important drivers of dysbiosis. It has recently been shown in vitro that, apart from classic antimicrobials, many other drugs have an extensive impact on human gut bacteria.^19^ A population‐based deep sequencing study of the faecal microbiome revealed that proton pump inhibitors (PPI) were associated with the most profound microbiome changes, followed by statins, antibiotics, laxatives and beta blockers.^20^ Nearly 50% of older adults take one or more medications that are not medically indicated and 45% of patients over the age of 75 take five or more drugs per day.^21^ The known consequences of polypharmacy and over‐medication are increased healthcare costs, adverse drug reactions, increased rates of drug‐drug interactions, decreased performance status of the patients, cognitive impairment, higher risk of falls and non‐compliance. Interventions to reduce polypharmacy are difficult to implement.^22^, ^23^

From the currently available data it is not known which of the above described factors may have the strongest impact on faecal microbiome composition in cirrhosis and would therefore be the most promising therapeutic goal.

We therefore conducted a systems biology analysis on a large dataset of cirrhotic patients of different aetiology and disease severity to analyse, which factors have the strongest influence on faecal microbiome composition and predicted metagenomics in patients with liver cirrhosis.

## 2 MATERIALS AND METHODS

### 2.1 Patients

We included faecal 16S microbiome sequencing data from cirrhotic patients recruited at the outpatient clinic of the Department of Gastroenterology and Hepatology, University Hospital of Graz, Austria who were screened for an intervention study^10^ between July 2012 and September 2013 in this post‐hoc analysis. All patients gave written informed consent. Diagnosis of cirrhosis was based on liver histopathological examinations, or a combination of clinical, radiological and/or labouratory features. Patients with a Child‐Pugh score of 12 or higher, alcohol consumption within 2 weeks prior to inclusion, active infection at screening, gastrointestinal haemorrhage within 2 weeks prior to inclusion, immuno‐modulating drugs, hepatic encephalopathy stage two or higher, renal failure (creatinine over 1.7 mg/dL), other severe diseases unrelated to cirrhosis, malignancy or pregnancy were excluded. Stool and blood samples analysed in this study were taken before patients received any study medication. The study protocol was approved by the institutional review board (ethics committee) in Graz (23‐096 ex 10/11), registered at http://clinicaltrials.gov (NCT01607528) and performed according to the declaration of Helsinki. The following characteristics were assessed as possible microbiome shaping factors: Age, sex, smoking status, aetiology of cirrhosis (alcohol, hepatitis C and other aetiologies; other aetiologies contained cholestatic liver disease, non‐alcoholic steatohepatitis, hepatitis B, hemochromatosis and Wilsons disease), severity of liver disease (Child‐Pugh grade and Model for End‐stage Liver Disease (MELD) score and the individual labouratory parameters albumin, bilirubin, hemoglobin, uric acid, creatinine, prothrombin time (international normalized ratio) as well as complications (presence of ascites, hepatic encephalopathy), nutritional status (Subjective Global Assessment (SGA)),^24^ comorbidities,^25^ drug intake (number of different drug classes and the following individual drug classes: proton pump inhibitors, beta blocker, other antihypertensives, diuretics, lactulose, antidiabetics, antidepressants, silymarin), intestinal permeability (lactulose/mannitol ratio, sucrose recovery, zonulin in stool, diamino‐oxidase (DAO) in serum), intestinal inflammation (calprotectin in stool) and systemic inflammation (C‐reactive protein (CRP), interleukin‐6, interleukin‐8, interleukin‐10, tumour necrosis factor (TNF)‐alpha, soluble CD 163 (sCD163), soluble Mannose receptor (sMR), each in plasma); neutrophil resting, priming and full burst; biomarkers of bacterial translocation (lipopolysaccharide [LPS], lipopolysaccharide binding protein [LBP], soluble CD14 (sCD14)).

### 2.2 Total DNA isolation, 16S library preparation, sequencing and analysis

Total DNA was isolated from frozen stool samples using MagnaPure LC DNA Isolation Kit III (Bacteria, Fungi) (Roche, Mannheim, Germany) according to manufacturer's instructions including mechanic and enzymatic lysis as described in Klymiuk et al 2017.^26^ For 16S rDNA sequencing hypervariable regions V1‐V2 were amplified in a target‐specific PCR (primers: 27F‐AGAGTTTGATCCTGGCTCAG; R357‐CTGCTGCCTYCCGTA) and amplification products were sequenced after indexing and purification on an Illumina MiSeq desktop sequencer (Illumina, Eindhoven, the Netherlands) according to published procedures.^26^, ^27^

### 2.3 Statistical analysis

For microbiome analysis, demultipexed FASTQ files were processed using Qiime2 tools implemented in Galaxy (https://galaxy.medunigraz.at). Denoising (removing primers, quality filtering, correcting errors in marginal sequences, removing chimeric sequences, removing singletons, joining paired‐end reads and dereplication) were done with DADA2.^28^ Taxonomy assignement was based on Silva 132 database release at 99% OTU level and trained using a Naïve Bayes classifier. After pre‐processing, an average of 59 369 reads per sample could be reached. All analyses, except diversity analysis, were done on an unrarefied feature table. For normalization Hellinger transformation was used. Rare taxa with a relative abundance of less than 0.01% across all samples were filtered. Chloroplast and cyanobacteria filtering was performed to remove contaminants. Alpha diversity analysis was performed using Chao1 on a rarefied feature table (sequencing depth 14 086). Beta diversity was analysed with Redundancy Analysis (RDA)^29^ based on Bray Curtis dissimilarity. Differentially abundant taxa were identified with Analysis of Composition of Microbiomes (ANCOM).^30^ As machine learning methods to select genera associated with the explanatory variables, LDA‐effect size (LEfSe)^31^ was used for categorical variables and Least Absolute Shrinkage and Selection Operator (LASSO) Regularized Regression^32^ for continuous variables. Network analysis was based on Spearman's rho associations between taxa and converting the pairwise correlations into dissimilarities to ordinate nodes in a two dimensional PCoA plot. Nearest Neighbour Propensity Score matching was performed without replacement based on logistic regression, using R 3.6.1^33^ package “MatchIt.”^34^, ^35^ The web‐based software Calypso version 7.14 (http://cgenome.net/calypso/) was used for analyses of microbiome data.^36^ For non‐microbiome analyses SPSS V25.0.0.1 (IBM, Armonk, NY, USA) was used (descriptive statistics, group comparisions, Spearman Rho correlation, Collinearity analysis) Visualization was performed in R 3.6.1^33^ package “ggplot2”.^37^ Sequencing data have been made publically available at the NCBI Sequence Read Archive (accession number PRJNA390475). A formal samples size calculation was not performed.

### 2.4 Labouratory measurements

Albumin, bilirubin, hemoglobin, uric acid, creatinine, prothrombin time and CRP were measured in the routine labouratory. LPS was detected in serum with an adapted protocol using HEK‐Blue hTLR4 reporter cells (Invivogen, Toulouse, France) as published previously.^38^ All other assays were handled according to manufacturers’ instructions. Enzyme linked immunosorbent assay were used to measure calprotectin, zonulin and serum DAO (Immundiagnostic AG, Bensheim, Germany), as well as LBP and sCD14 (Hycult biotechnology, Uden, the Netherlands). sCD163 and sMR in plasma samples were measured by an in‐house sandwich ELISA using a BEP‐2000 ELISA‐analyser (Dade Behring) as previously described.^10^, ^39^, ^40^

Cytokines (interleukin‐6, interleukin‐8, interleukin‐10, TNF‐alpha) were measured with ProcartaPlex (eBioscience, Vienna, Austria). Neutrophil oxidative burst was measured by flow cytometry using a commercially available kit (Glycotope, Heidelberg, Germany).

## 3 RESULTS

### 3.1 Study cohort

Microbiome sequence data of 88 cirrhotic patients were analysed. Patient characteristics are presented in Table 1. We analysed the impact of age, sex, smoking status, aetiology of cirrhosis, severity of liver disease, comorbidities, nutritional status, drug intake, intestinal permeability, intestinal and systemic inflammation on faecal microbiome composition. Drug classes that were taken by more than 15% of the study population were included with the exception of lactulose, which was used by only 11% of the study population but was included into univariate analysis due to its supposed microbiome modulating properties. Antibiotics (chinolons) for prophylaxis of spontaneous bacterial peritonitis were only taken by 2 out of 88 patients and rifaximin was not taken by any patients. Moderate malnutrition was more frequent in hepatitis C cirrhosis (43.8%) compared to alcoholic cirrhosis (19.1%) or other aetilologies (4%, *P* = .006). No other significant differences between categorical variables were found. Spearman correlation showed a weak, but significant correlation between CRP and disease severity (Spearman Rho *r* = 0.380, *P* < .001). No significant correlation between other variables was found. Testing for collinearity showed that all combinations of explanatory variables had a VIF <1.5, excluding a strong codependence.

**Table 1:** Patient characteristics

	Cirrhosis (n = 88)
Age (years)	58 (56; 61)
Sex (m/f)	62/26 (70.5%/29.5%)
Aetiology (alcohol/hepatitis C/other)	47/16/25 (53.4%/18.2%/ 28.4%)
Child‐Pugh Grade (A/B/C)	66/20/2 (75%/22.7%/2.3%)
MELD score	10 (9;12)
SGA Grade (adequate/moderate malnutrition)	71/17 (80.7%/19.3%)
PPI (y/n)	48/40 (54.5%/45.5%)
Lactulose (y/n)	9/79 (10.2%/89.8%)
Albumin (mg/dl)	4.2 (4.0; 4.3)
Bilirubin (mg/dl)	1.2 (1.0; 3.0)
CRP (mg/l)	2.5 (1.9; 3.0)
Creatinine (mg/dl)	0.84 (0.79;0.9)
INR	1.24 (1.19; 1.27)

Note

Data are given as absolute numbers and percentage, or median and 95% confidence interval (lower; upper).

Abbreviations: CRP, C‐reactive protein; INR, international normalized ratio; MELD, model of end stage liver disease; PPI, proton pump inhibitor; SGA, subjective global assessment.

### 3.2 Differences in microbiome composition between groups (beta diversity)

**Figure 1:**
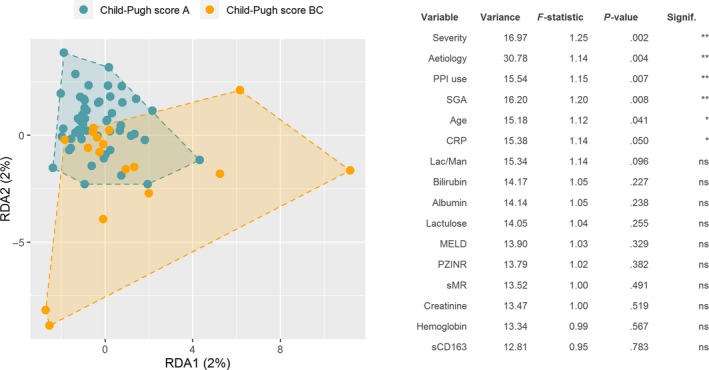
Multivariate redundancy analysis (RDA+) based on Bray‐Curtis dissimilarity. Disease severity was chosen as grouping variable due to the lowest *P*‐value on univariate analysis. The effect of the other explanatory variables is also included in the model. The table shows the results of multivariate redundancy analysis for variables with significant effects on univariate analysis

Univariate RDA revealed that severity of liver disease (assessed by Child‐Pugh and MELD score), aetiology, PPI use, lactulose use, nutritional status (assessed by SGA), age, lactulose/mannitol ratio, albumin, bilirubin, CRP, hemoglobin, creatinine, INR, sCD163 and sMR were significant explanatory variables for microbiome composition (*P* < .1). These variables were included into a multivariate model, where severity of disease, aetiology, PPI use, nutritional status, age and CRP remained as significant explanatory variables (*P* < .05; Figure 1).

### 3.3 Species diversity (alpha diversity) and taxonomic differences

We analysed differences in alpha diversity and taxonomic composition in relation to the six variables (severity of disease, aetiology, PPI use, nutritional status, age and CRP) that significantly affected beta diversity of the stool microbiome composition.

For alpha diversity analyses the feature table was rarefied to 14 086 reads. There were no changes in alpha diversity in samples from patients with Child‐Pugh A vs Child‐Pugh B/C cirrhosis, with different aetiologies, between PPI use or non‐use, or between patients with adequate nutritional status compared to moderate malnutrition. Furthermore, age and CRP did not correlate with alpha diversity (Chao1).

**Figure 2:**
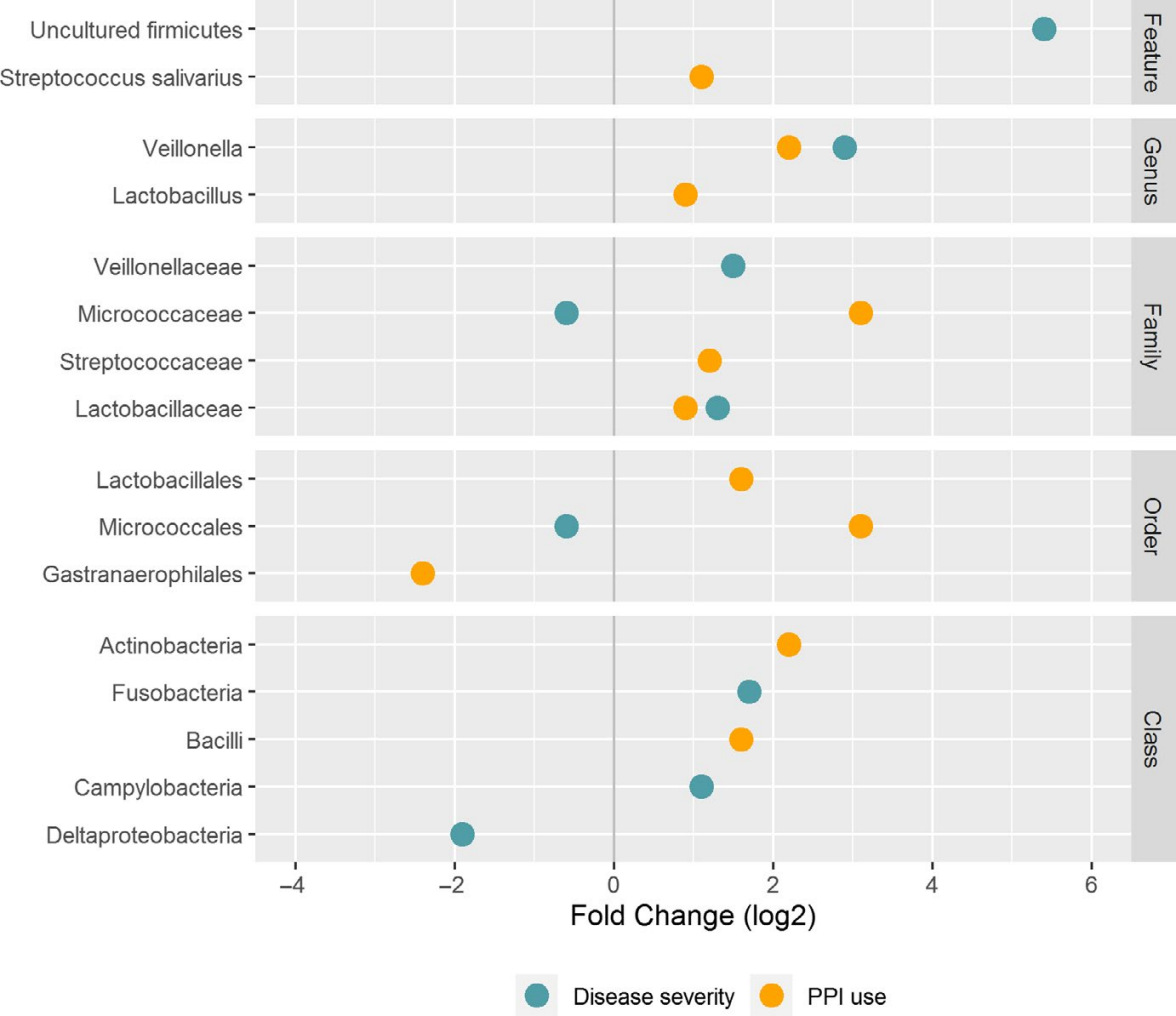
Differentially abundant taxa for disease severity groups and PPI use/non‐use based on ANCOM analysis. ANCOM analysis does not report *P*‐values. All features/genera/families/orders/classes shown in this graph are significantly different between the groups

**Figure 3:**
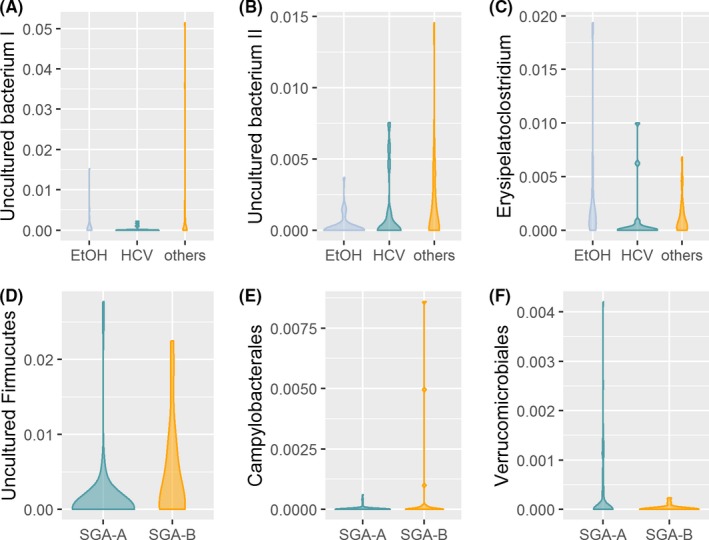
Differentially abundant taxa for aetiology (A‐C) and nutritional status (D‐F) based on ANCOM analysis. ANCOM analysis does not report *P*‐values. All features/genera/families/orders/classes shown in this graph are significantly different between the groups

**Figure 4:**
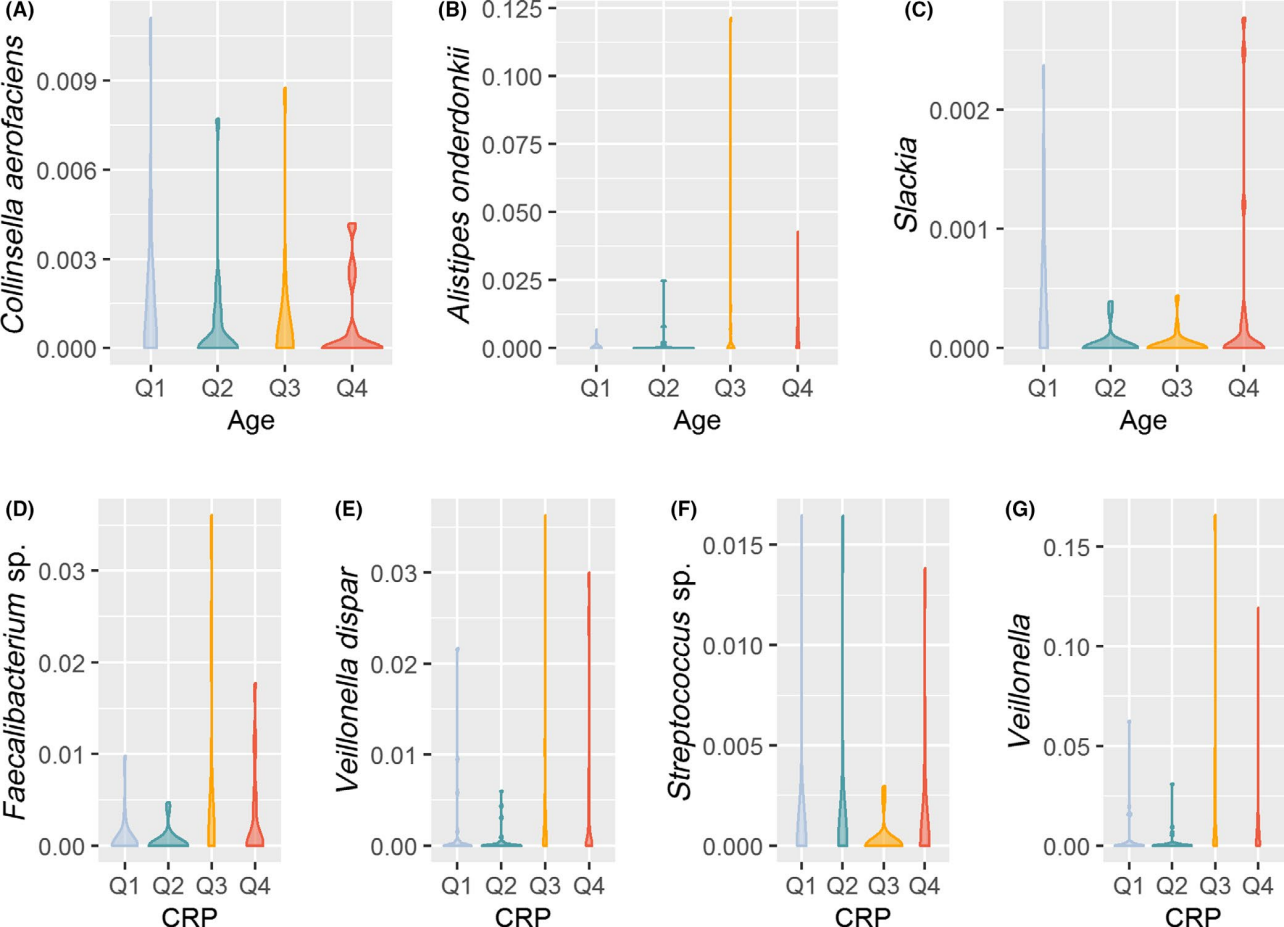
Differentially abundant taxa for age (A‐C) and CRP (D‐G) based on ANCOM analysis. ANCOM analysis does not report *P*‐values. All features/genera/families/orders/classes shown in this graph are significantly different between the groups

Analysis of Composition of Microbiome revelead that one uncultured bacterium of the phylum Firmicutes, the genus *Veillonella*, the families *Lactobacillaceae* and *Veillonellaceae* and the classes Campylobacteria and Fusobacteria were more abundant in Child‐Pugh B/C cirrhosis whereas the family *Micrococcaceae*, the order *Micrococcales* and the class Deltaproteobacteria were more abundant in patients with Child‐Pugh A cirrhosis. (Figure 2) PPI user showed a higher abundance of the feature *Streptococcus salivarius*, the genera *Lactobacillus* and *Veillonella*, the families *Lactobacillaceae, Micrococcaceae* and *Streptococcaceae*, the orders Lactobacillales and Micrococcales and the classes Actinobacteria and Bacilli, whereas the order Gastranaerophilales was higher abundant in PPI non‐users. (Figure 2) Patients with alcoholic cirrhosis had a higher abundance of the genus *Erysipelatoclostridium*. Patients with “other” aetiologies of liver cirrhosis had a higher abundance of one uncultured bacterium of the family *Lachnospiraceae* and one uncultured bacterium of the genus *Blautia* on feature level. No differences at higher taxonomic levels were found for aetiology of cirrhosis. (Figure 3A‐C) Patients with adequate nutrition showed lower abundances of an uncultured bacterium of the phylum Firmicutes and the order Campylobacterales. In addition, a higher abundance of the order Verrucomicrobiales compared to moderate malnutrition was found (Figure 3D‐F). The feature *Collinsella aerofaciens* and the genus *Slackia* showed a decreasing abundance with increasing age whereas the feature *Alistipes onderdonkii* increases with age. On higher taxonomic levels no age‐dependent differences were found (Figure 4A‐C). The third and fourth quartile of CRP levels was associated with higher abundance of the features *Faecalibacterium *sp.*, Veillonella dispar* and of the genus *Veillonella. Streptococcus species* was lowest in the third quartile of CRP levels compared to the other quartiles*.* No differences on higher taxonomic levels were found. (Figure 4D‐G).

### 3.4 Machine learning and network analysis

**Figure 5:**
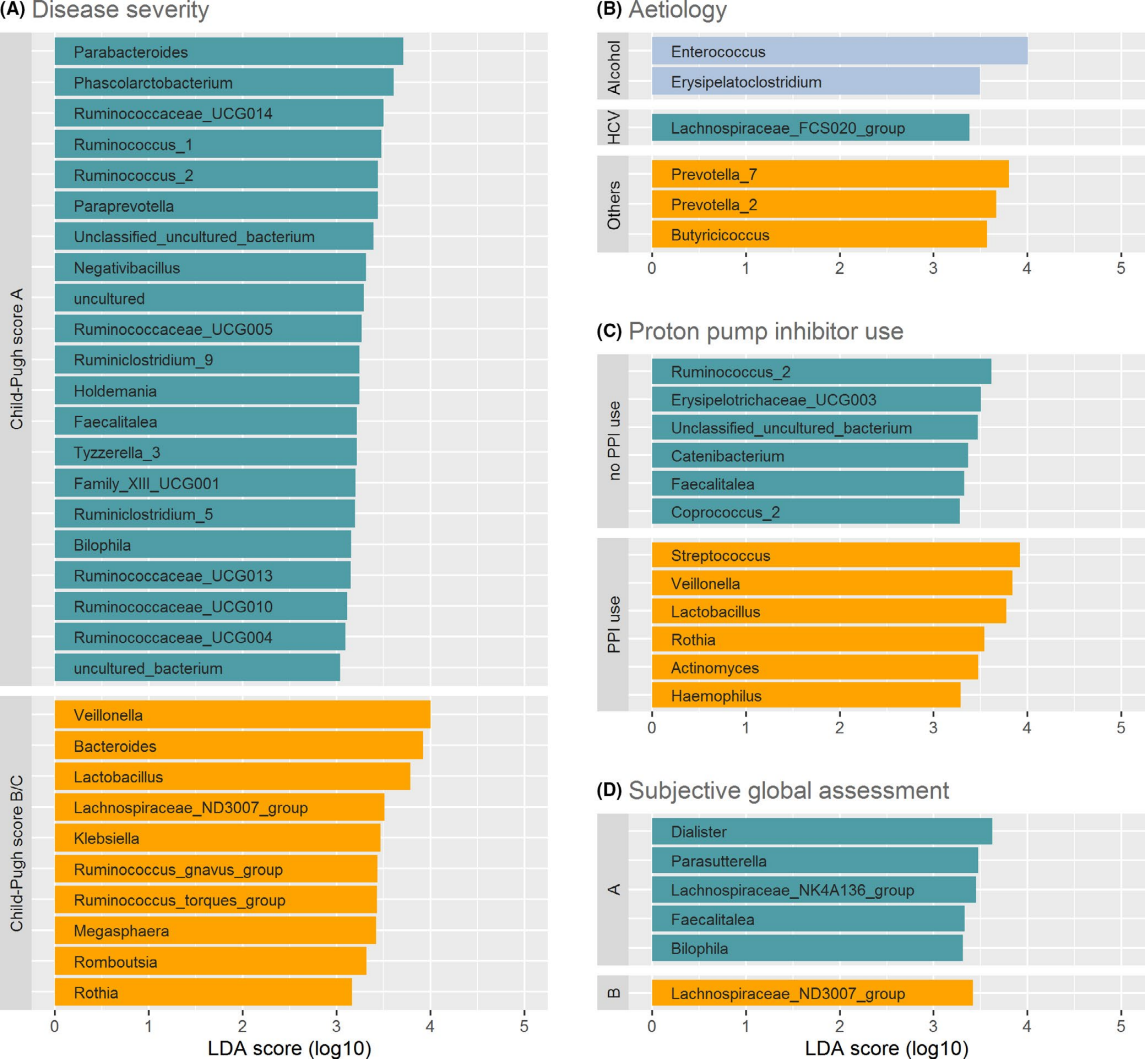
Most differentially abundant taxa selected by Linear discriminant analysis Effect Size (LEfSe) for (A) Disease severity, (B) aetiology, (C) PPI use/non‐use, (D) nutritional status

To further understand the association of microbiome composition with the factors that were identified to significantly influence beta diversity, we used supervised machine learning algorithms as a feature selection method on genus level. LEfSe identified 21 genera to be associated with Child‐Pugh A cirrhosis and 10 genera to be associated with Child‐Pugh B/C cirrhosis. (Figure 5A) Among the genera associated with Child‐Pugh B/C cirrhosis, oral bacteria such as *Veillonella, Lactobacillus and Rothia* and potential pathogens such as *Klebsiella* were found. Hepatitis C was associated with *Lachnospiraceae FCS020 group*, alcoholic cirrhosis with *Enterococcus* and *Erysipelatoclostridium,* and other aetiologies with two *Prevotella* genera and *Butyricicoccus*. (Figure 5B) PPI use was associated with six genera, all of which are either oral commensal bacteria (*Veillonella, Streptococcus, Lactobacillus, Rothia*) or potential pathogens (*Actinomyces, Haemophilus*). PPI non‐use was associated with *Ruminococcus, Erysipelotrichaceae, Catenibacterium, Faecalitalea, Coprococcus* and one unclassified uncultured bacterium. (Figure 5C) Moderate malnutrition was associated with *Lachnospiraceae ND3007 group,* whereas adequate nutritional status was associated with *Dialister, Parasutterella, Lachnospiraceae NK4A136 group*, *Faecalitalea* and *Bilophila*. (Figure 5D) No genera were identified with LASSO to be associated with age or CRP levels.

**Figure 6:**
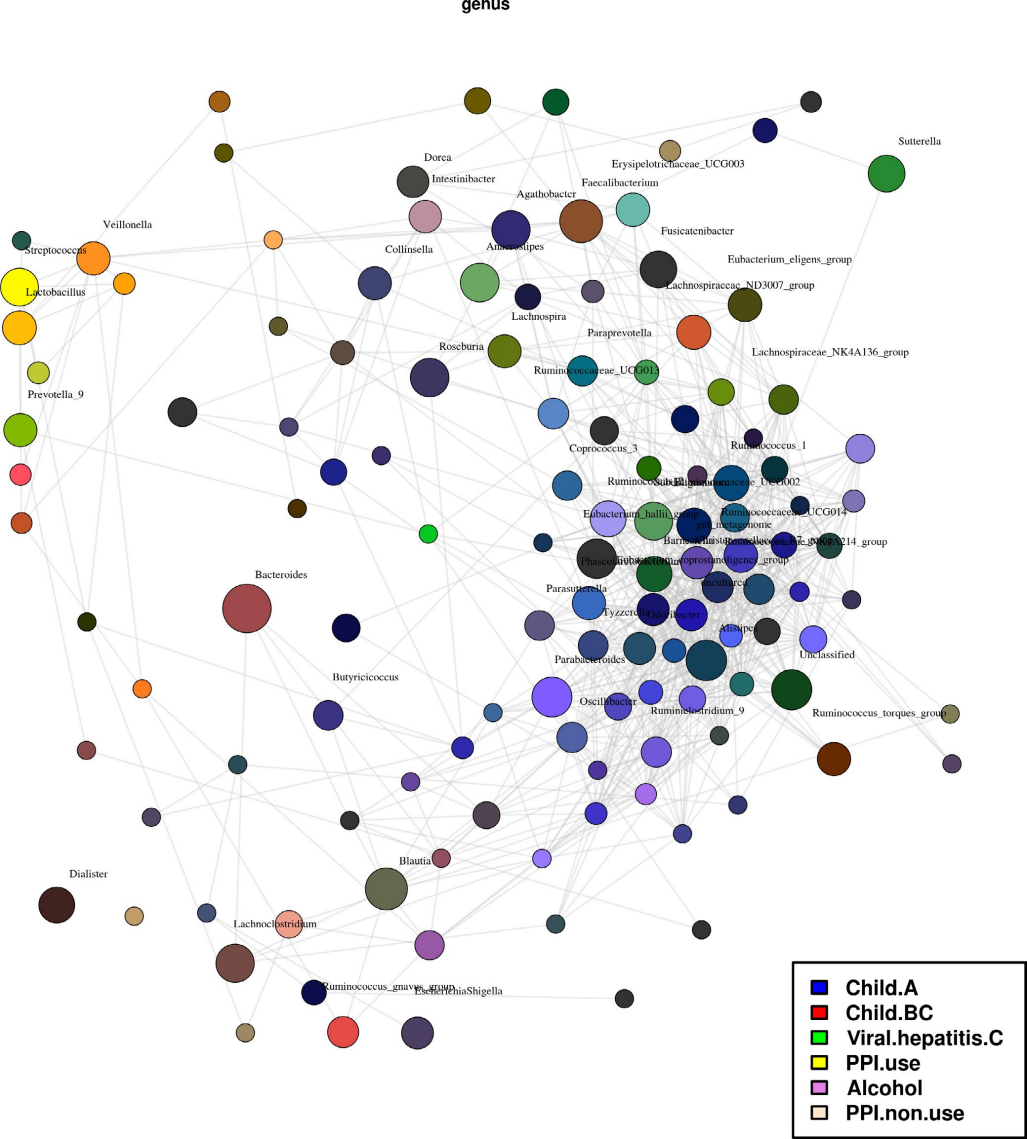
Network analysis to identify associations between bacteria and selected host variables. Taxa and explanatory variables are represented as nodes, taxa abundance as node size, and edges represent positive and negative associations. Nodes (genera) are coloured based on their association with selected host variables (disease severity, PPI use/non‐use and aetiology). A, Whole cohort (n = 88). B, Child A cirrhosis (n = 67) and © Child B/C cirrhosis (n = 21)

To visualize the relation of these significant influencing factors we performed a network analysis. Network analysis including severity, PPI use and aetiology as explanatory variables showed some overlaps but also some distinct genera that were only associated with one of the explanatory variables. (Figure 6) Although PPI use is statistically equally frequent in Child‐Pugh B/C cirrhosis (Fisher exact *P* = .085) compared to Child‐Pugh A cirrhosis and collinearity analysis shows no collinearity (VIF = 1.250), the largest overlap is found for genera associated with Child‐Pugh B/C cirrhosis and PPI use (orange colour). About 71% of Child‐Pugh B/C patients use PPI compared to 49% in the Child‐Pugh A group. PPI user have a significantly higher MELD score compared to PPI non‐users (12 vs 10, *P* = .011), making it challenging to distinguish between PPI induced and severity induced microbiome changes in cirrhosis in this dataset.

To distinguish the effect of severity and PPI use better, we performed the following analyses on subgroups of the initial dataset. To balance the confounding influence of aetiology, PPI use, nutritional status, age and CRP when comparing disease severity stages (Child A vs ChildB/C) we performed nearest neighbour propensity score matching without replacement based on logistic regression. This resulted in a dataset of 21 Child B/C patients and 21 matched Child A patients with a median matching distance of 0.29 (95% CI interval 0.24; 0.35). After propensity score matching, disease severity, aetiology, PPI use and age were still significant explanatory variables of microbiome composition on multivariate RDA whereas CRP and nutritional status did not influence microbiome composition significantly any more (see Figure S1). Feature selection by LEfSe in the propensity score matched cohort showed comparable results as obtained from the original, non‐matched dataset, indicating that the microbiome effects are true effects and not caused by other confounders (see Figure S2). To confirm the influence of PPI use independent of severity of liver disease, we additionally performed a subgroup analysis for Child A cirrhosis and Child B/C cirrhosis separately. When performing multivariate RDA analysis separately for severity groups, we observed that PPI use (*P* = .035) in Child A cirrhosis and aetiology (*P* = .015), PPI use (*P* = .038) and age (*P* = .014) in the Child B/C group were still predictive for microbiome composition. Due to low sample size in the subgroups the results have to be interpreted with caution.

## 4 DISCUSSION

Faecal microbiome composition has been associated with various diseases, but not only disease severity and comorbidities, but also other factors such as medication, diet or lifestyle habits may influence its composition. It is therefore important to discern the effect of different influencing variables as a first step from association to causality. We performed a system biology analysis of a well‐characterized cohort of patients with liver cirrhosis and were able to show by multivariate RDA that severity and aetiology of liver disease, PPI use, nutritional status, age and CRP levels were significant explanatory variables for faecal microbiome composition. Although we find some taxonomic overlaps especially between severity and PPI use on network analysis, we could show that the effects of disease severity, aetiology, PPI use and age are independent factors influencing microbiome composition also in subgroup analyses.

Changes in faecal microbiome composition have been associated with liver disease since more than 35 years, long before sequencing techniques became available.^41^ When 16S sequencing techniques emerged, stool microbiome composition in liver disease was studied showing compositional alterations throughout all levels of taxonomy in liver cirrhosis.^3^, ^42^ However both of these studies did not associate compositional changes with potentially important influencing factors such as aetiology of cirrhosis or drug intake in a systematic way. The strength of our cohort is the detailed characterization of the participants, that allows to fill this knowledge gap on the association of different factors with faecal microbiome composition in cirrhosis and the use of ANCOM and LEfSE as specialized bioinformatics methods to study microbiome composition.

Severity of liver disease was shown to impact microbiome composition. A positive correlation between Child‐Pugh score and *Streptococcaceae* as well as a negative correlation with *Lachnospiraceae* was described in the study by Chen et al Several positive and negative correlations between liver function and species abundance were reported in the study by Qin et al without describing more details on these associations.^3^, ^42^ Data on concomidant drug intake is missing in both studies, leading to scientific discussions and the need for further reserach^43^, ^44^ In subsequent studies strong associations of microbiome changes with hepatic encephalopathy were shown.^8^, ^45^ Our analysis demonstrates that disease severity, measured by composite scores (Child‐Pugh and MELD) as well as some of the individual parameters of both scores (albumin, bilirubin, creatinine, INR) are significant explanatory variables for microbiome composition in univariate analysis. Child‐Pugh score also remained significant in multivariate RDA. Higher Child‐Pugh classes (B and C) were associated with distinct changes in microbiome composition related to an increase in oral bacteria and potential pathogens. On family level we found a higher abundance of *Lactobacillaceae* and *Veillonellaceae* and a lower abundance of *Micrococcaceae* in Child‐Pugh B/C patients which is in line with previously published data.^3^, ^8^, ^42^, ^45^ However, it is still not fully elucidated, whether these changes are driven by disease severity itself or by other influencing factors.

Cirrhosis is a complex disease requiring long‐term drug treatment with several drug classes. Many medically approved drugs influence microbiome composition.^19^ In liver cirrhosis, PPI use has been described to alter microbiome composition, increase the rate of complications and negatively impact prognosis.^5^, ^46^, ^47^, ^48^, ^49^ We recently expanded this knowledge by describing the consequences of PPI‐induced dysbiosis and oralization of the faecal microbiome on inflammation, intestinal permeability and outcome in cirrhosis.^11^ In the present study PPI use also had a strong impact on the faecal microbiome, being associated with an increased abundance of oral bacteria and potential pathogens, such as *Streptococcus species* and *Veillonella*. Although PPI use and disease severity were partially linked in our cohort, both were still independent factors influencing microbiome composition in subgroup analyses, however low sample size in this analysis weakens the explanatory power.

Also other drugs commonly administered in cirrhosis, may impact the faecal microbiome. However, in our cohort, other drugs influenced microbiome composition to a lesser extent. We were interested in the effect of lactulose on the cirrhotic microbiome, since available data are conflicting, showing no major alterations of the faecal microbiome between lactulose user and non‐user, but withdrawal of lactulose leads to the loss of beneficial species.^16^, ^50^, ^51^ In our cohort only 11% of the cirrhotic patients used lactulose. On univariate analysis we found that lactulose had a weak, but significant, effect on microbiome composition, which did not remain significant in multivariate analysis. Therefore our data support the notion that lactulose has no major effect on the taxonomic composition of the faecal microbiome in cirrhosis and may therefore exert its function through functional microbiome changes. Other drug classes did not have a major impact on microbiome composition.

Liver cirrhosis is also a heterogeneous disease from an aetiological perspective. Aetiology of liver disease as a factor to explain differences in microbiome composition has already been studied. Bajaj et al describe alcohol and non‐alcoholic steatohepatitis driven changes in microbiome composition.^45^ Hepatitis C alters faecal microbiome which has been implicated in the pathogenesis of HCV‐induced chronic liver disease. However, Hepatitis C induced dysbiosis seems to be stable over different disease stages.^52^ Chronic cholestatic diseases such as primary biliary and primary sclerosing cholangitis are also associated with distinct changes in microbiome composition.^53^ Although aetiology of liver disease was identified as a significant factor by multivariate RDA in our data set, the taxonomic differences of the microbiomes from different aetiologies were surprisingly small in our cohort. Patients with alcoholic cirrhosis had a higher abundance of the genus *Erysipelatoclostridium* and the group of patients with “other” aetiologies of liver cirrhosis had a higher abundance of two yet uncultured bacteria of the family *Lachnospiraceae* and of the genus *Blautia*. Since liver cirrhosis aetiology is varying in different geographical regions^54^ and geographical region itself is a factor that impacts diversity and composition of the microbiome,^55^ also the origin of the study population has to be taken into account. Our patient cohort consists only of Caucasians living in the same region, which is a likely explanation for the relatively similar microbiome composition in different aetiologies. Bajaj et al have shown that cirrhotic patients from Turkey, compared to patients from the USA, differ in aetiology and dietary habits, which resulted in a higher microbial diversity in Turkish cirrhotic patients.^14^ We also analysed dietary habits in our patient cohort and only found one significant difference of questionable relevance. Patients with hepatitis C virus infection were more likely to consume muesli on a regular basis compared to alcoholic cirrhotic patients (62% vs 32%, *P* < .01) 56

Furthermore age impacts on microbiome composition, however, the specific changes of the ageing microbiome are unknown and inconsistent. Data on microbial diversity are conflicting and seem to depend on confounding factors and analysis techniques. A loss of *Bifidobacteria*, *Lactobacilli*, *Clostridium Cluster XIVa, Akkermansia muciniphila* and *Faecalibacterium prausnitzii* and an increase of *Escherichia coli* species has been observed.^57^ *Collinsella aerofaciens, Alistipes onderdonkii* and the genus *Slackia* were differentially abundant between age groups in our cirrhosis cohort. However, none of these have been previously described to be associated with age related changes in microbiome composition. *Collinsella aerofaciens* has been associated with diet and intestinal inflammatory diseases,^58^, ^59^, ^60^ Slackia with equol production,^61^ whereas *Alistipes onderdonkii* has not been associated with any human condition yet.^62^

Nutritional status is an important prognostic factor in liver cirrhosis. Especially sarcopenia is a frequent complication of malnutrition in cirrhosis and is associated with adverse outcome.^63^, ^64^ Assessing nutritional status in cirrhosis can be challenging. We used the well‐established SGA, that is also recommended by the European Association for the Study of the Liver^65^ to differentiate between well nourished and moderately malnourished patients. In our cohort of mostly compensated cirrhotic patients we did not have any severely malnourished patients. We found a higher abundance of the order *Campylobacterales* in moderately malnourished cirrhotics. *Campylobacterales* have been associated with malnutrition in children.^66^, ^67^ The order *Verrucomicrobiales* was enriched in adequately nourished cirrhotic patients. So far only one species of this order has been described in human faeces: *Akkermansia muciniphila*. A loss of *Akkermansia muciniphila* has been associated with metabolic diseases and the abundance may be influenced by diet.^68^

Changes in microbiome composition in cirrhosis impact intestinal barrier function and lead to intestinal and systemic inflammation due to translocation of bacterial products to the liver and also to the systemic circulation. This concept of a crosstalk between gut, liver and immune system – the so called gut‐liver axis—is widely implicated in the pathogenesis of liver disease and a promising therapeutic target.^69^, ^70^ We assessed a panel of biomarkers of the gut–liver axis in our cohort and CRP as a marker of inflammation was found to be a significantly explanatory variable in the multivariate analysis, however the consistency of the effect remains unclear in our dataset. CRP is a well‐known biomarker in liver cirrhosis and is predictive for complications and outcome.^71^, ^72^ Increased abundance of *Veillonella* and *Streptococcus* species but also *Faecalibacterium* species were associated with higher CRP levels. While the former support the link between microbiome composition and inflammation in liver cirrhosis, *Faecalibacterium* species are usually associated with anti‐inflammatory properties, making a firm conclusion difficult.

Interestingly, gender did not impact on microbiome composition in our study. In studies in obese individuals, gender seems to cause taxonomic differences, whereas data on changes on alpha and beta diversity are still conflicting.^73^, ^74^ In liver cirrhosis, gender differences have not been associated with changes in beta diversity so far. The male predominance in liver cirrhosis may be a reason for difficulties to detect consistent gender differences.

In summary our cross sectional system biology study shows that disease severity and PPI use are the main factors explaining variation of the faecal microbiome in cirrhosis. Aetiology of liver disease, age, nutritional status and inflammation (CRP levels) are further explanatory variables. The limitation of our study is the single‐center design that does not allow to account for geographical differences in the microbiome composition, the cross sectional design that does not allow to draw any conclusions on causality as well as the low sample size in the subgroup analyses. The strength of our study is the thorough characterization of our study patients, that allows detailed analysis of influencing variables. For future studies, we strongly suggest to increase sample size and expand the minimal set of metadata as suggested by the IHMS consortium^75^ to include detailed information on disease aetiology, disease severity, drug intake and also information on further essential biomarkers, depending on the disease studied. This will open up new paths to understand the crosstalk between the faecal microbiome and the human body in disease states.

### 4.1 Lay summary

The composition of gut bacteria—the gut microbiome—is altered in many diseases. In chronic liver diseases, such as liver cirrhosis, the gut microbiome is severely disturbed. We were able to show in this study which factors explain this disturbed microbiome composition. These factors were: The cause of liver disease, the severity of liver disease, intake of acid‐blockers (proton pump inhibitors), age and inflammation. Our study also shows the importance of collecting sufficient data on the disease and drug intake to be able to assess the effects of different factors on the gut microbiome.

## CONFLICT OF INTEREST

None of the authors declares any conflict of interest.
