# Supplementary material for: Disease severity and proton pump inhibitor use impact strongest on faecal microbiome composition in liver cirrhosis
Source: Liver Int. 2020 Jan 24;40(4):866–77. doi: 10.1111/liv.14382 (PMC7187411; doi:10.1111/liv.14382)
Supplement: Supplementary file 1 [file LIV-40-866-s001.docx]

**Disease severity and proton pump inhibitor use impact strongest on faecal microbiome composition in liver cirrhosis**

Vanessa Stadlbauer, MD,^1,2*^ Irina Komarova, MD,^1^ Ingeborg Klymiuk, PhD,^3^ Marija Durdevic, MSc,^3,4^ Alexander Reisinger, MD,^5^ Andreas Blesl, MD^1^, Florian Rainer, MD^1^, Angela Horvath, PhD,^1,2^

Supplementary figure 1

Figure S1: Multivariate redundancy analysis (RDA+) based on Bray-Curtis dissimilarity in the subgroup of Child B/C cirrhosis (n=21) and propensity score matched Child A cirrhotic patients (n=21). Disease severity was chosen as grouping variable. The effect of the other explanatory variables is also included in the model. The table shows the results of multivariate redundancy analysis in the propensity score matched cohort.

Supplementary Figure 2

Figure S2: Most differentially abundant taxa selected by Linear discriminant analysis Effect Size (LEfSe) in the propensity score matched cohort (21 Child B/C and 21 matched Child A patients) for A: Disease severity, B: aetiology, C: PPI use/non-use

With LASSO no genera associated with age could be found.
